# Exosome-mediated delivery of gga-miR-20a-5p regulates immune response of chicken macrophages by targeting IFNGR2, MAPK1, MAP3K5, and MAP3K14

**DOI:** 10.5713/ab.22.0373

**Published:** 2023-01-11

**Authors:** Yeojin Hong, Jubi Heo, Suyeon Kang, Thi Hao Vu, Hyun S. Lillehoj, Yeong Ho Hong

**Affiliations:** 1 Department of Animal Science and Technology, Chung-Ang University, Anseong 17546, Korea; 2 Animal Biosciences and Biotechnology Laboratory, Agricultural Research Services, United States Department of Agriculture, Beltsville, MD 20705, USA

**Keywords:** Apoptosis, Exosomes, MAPK Signaling Pathway, miRNAs, miR-20a-5p

## Abstract

**Objective:**

This study aims to evaluate the target genes of gga-miR-20a-5p and the regulated immune responses in the chicken macrophage cell line, HD11, by the exosome-mediated delivery of miR-20a-5p.

**Methods:**

Exosomes were purified from the chicken macrophage cell line HD11. Then, mimic gga-miR-20p or negative control miRNA were internalized into HD11 exosomes. HD11 cells were transfected with gga-miR-20a-5p or negative control miRNA containing exosomes. After 44 h of transfection, cells were incubated with or without 5 μg/mL poly(I:C) for 4 h. Then, expression of target genes and cytokines was evaluated by quantitative real-time polymerase chain reaction.

**Results:**

Using a luciferase reporter assay, we identified that gga-miR-20a-5p directly targeted interferon gamma receptor 2 (IFNGR2), mitogen-activated protein kinase 1 (MAPK1), mitogen-activated protein kinase kinase kinase 5 (MAP3K5), and mitogen-activated protein kinase kinase kinase 14 (MAP3K14). Moreover, the exosome-mediated delivery of gga-miR-20a-5p successfully repressed the expression of *IFNGR2*, *MAPK1*, *MAP3K5*, and *MAP3K14* in HD11 cells. The expressions of interferon-stimulated genes (MX dynamin like GTPase 1 [*MX1*], eukaryotic translation initiation factor 2A [*EIF2A*], and oligoadenylate synthase-like [*OASL*]) and proinflammatory cytokines (interferon-gamma [IFNG], interleukin-1 beta [IL1B], and tumor necrosis factor-alpha [TNFA]) were also downregulated by exosomal miR-20a-5p. In addition, the proliferation of HD11 cells was increased by exosomal miR-20a-5p.

**Conclusion:**

The exosome-mediated delivery of gga-miR-20a-5p regulated immune responses by controlling the MAPK and apoptotic signaling pathways. Furthermore, we expected that exosomal miR-20a-5p could maintain immune homeostasis against highly pathogenic avian influenza virus H5N1 infection by regulating the expression of proinflammatory cytokines and cell death.

## INTRODUCTION

Exosomes are cell membrane-derived small vesicles with diameters ranging from 30 to 150 nm that are generated by various cell types in biological fluids, such as plasma, lymph, saliva, semen, urine, and cerebrospinal fluid [1,2]. Exosomes contain various proteins and nucleic acids originating from “mother cells”, which are transmitted to recipient cells [3,4]. Particularly, exosomal microRNAs (miRNAs) regulate the gene expression of recipient cells by repressing and degrading their target mRNAs [5,6]. MiRNAs are small non-coding RNAs that are 18 to 23 nucleotides long that bind to the 3′-untranslated regions (UTRs) of the target mRNA complementary sequences. The miRNAs then repress and degrade the target mRNAs by forming an RNA-induced silencing complex and inhibiting mRNA translation [7]. Exosomal miRNAs derived from various cell types, such as immune cells, cancer cells, and stem cells, have been demonstrated to regulate immune responses, cell migration, metastasis, proliferation, and invasion by repressing target gene translation [8–10].

Recently, studies have been conducted on exosomes as a gene delivery vehicle for molecules, such as RNA or DNA [11–15]. Exosomes as “nature’s delivery system” have several advantages. Because exosomes are produced from host cells, they have low toxicity and immunogenicity and are easily internalized due to their exosomal host cell-derived membranes [16,17]. Moreover, exosomal membranes protect their nucleic acid contents from nuclease degradation [11].

MiR-20a, a member of the miR-17 family, is known as a regulator of immune responses [18]. For example, the expression of miR-20a-5p was also downregulated by stimulation of toll-like receptor 4 (TLR4) and TLR2 ligands in rheumatoid arthritis fibroblast-like synoviocytes [19]. Furthermore, miR-20a-5p decreased the expression of cytokines, such as interleukin (IL)-6, C-X-C motif chemokine ligand 10 (CXCL10), IL-1β, and tumor necrosis factor alpha (TNF-α), in macrophages. Moreover, the downregulated expression of miR-20a-5p by *Mycobacterium tuberculosis* induced the apoptosis of human macrophages by regulating c-Jun N-terminal kinase 2 (JNK2) [20]. MiR-20a-5p expression was downregulated in CD4+ T cells derived from patients with Vogt-Koyanagi-Harada disease and upregulation of miR-20a-5p suppressed the production of IL-17 by targeting oncostatin M and C-C motif chemokine ligand 1 [21].

In our previous study, the expression of gga-miR-20a-5p was down-regulated in exosomes derived from highly pathogenic avian influenza virus (HPAIV) H5N1-infected chickens compared with exosomes of non-infected chickens, and 32 immune-related genes were predicted as gga-miR-20a-5p targets [22]. However, there are limited studies about the regulatory function of miR-20a-5p in chickens. Therefore, in this study, we evaluated the target genes of gga-miR-20a-5p and demonstrated the immunomodulatory effects in a chicken macrophage cell line by the exosome-mediated delivery of miR-20a-5p.

## MATERIALS AND METHODS

### Chicken cell line culture and purification of exosomes

The chicken macrophage cell line HD11 [23] was maintained at Roswell Park Memorial Institute (RPMI) in 1640 medium (Thermo Fisher Scientific, Waltham, MA, USA) containing 100 IU/mL penicillin, 100 mg/mL streptomycin, and 10% fetal bovine serum (Thermo Fisher Scientific, USA) in a humidified incubator with 5% CO_2_ at 41°C. To purify exosomes, HD11 cells (1.0×10^7^) were seeded in three 100-mm cell culture dishes (SPL Life Sciences, Pocheon, Korea) in exosome-depleted RPMI 1640 medium containing 100 IU/mL penicillin, 100 mg/mL streptomycin, and 10% exosome-depleted fetal bovine serum (#EXO-FBSHI-250A-1; System Bioscience, Palo Alto, CA, USA) and incubated for 24 h. The cell culture supernatant was collected to purify exosomes using the ExoQuick-TC kit (#EXOTC50A-1; System Biosciences, USA) according to the manufacturer’s protocol. The concentration of the purified exosomes was measured using a Pierce bicinchoninic acid protein assay kit (Thermo Fisher Scientific, USA) according to the manufacturer’s protocol. For the characterization of exosomes, their particle size was measured using a nanoparticle analyzer (SZ-100; Horiba, Kyoto, Japan). Furthermore, western blotting was performed using antibodies against CD9 (#13174; Cell Signaling Technology, Danvers, MA, USA) and CD81 (#56039; Cell Signaling Technology, USA), according to previously described methods [24].

### Transfection of mimic miRNA into exosomes

We loaded miRNA to exosomes as described by Kim et al [25] with a slight modification. Mimic gga-miR-20a-5p and miR-NC (negative control miRNA) were synthesized by Bioneer (Daejeon, Korea). The sequence of the mimic gga-miR-20a-5p was 5′-UAAAGUGCUUAUAGUGCAG GUAG-3′ and that of the miR-NC was 5′-UUCUCCGAA CGUGUCACGU-3′. For loading of the miRNAs into the exosomes, 20 pmol of mimic gga-miR-20-5p or miR-NC were diluted in 100 μL of Opti-MEM I Reduced Serum Medium (Gibco, Waltham, MA, USA) and 6 μL of Lipofectamine RNAiMAX Transfection Reagent (Invitrogen, San Diego, CA, USA) was diluted in 100 μL of Opti-MEM I Reduced Serum Medium. Diluted miRNA solutions were added to the diluted Lipofectamine RNAiMAX and incubated for 20 min at room temperature. Next, 100 μg of purified exosomes were incubated with miRNA-Lipofectamine RNAiMAX complexes for 6 h in a humidified incubator with 5% CO_2_ at 41°C. Then, the complexes were filtered with a 100 kDa Amicon Ultra-0.5 Centrifugal Filter (Merck Millipore, Burlington, MA, USA) to remove un-loaded miRNA in the exosomes. Afterward, 1.2×10^6^ cells/well of HD11 cells were plated in 12-well plates (SPL, Korea) containing 1.0 mL of exosome-depleted RPMI 1640 medium and incubated with the concentrated exosomes. After 44 h of incubation, cells were incubated with or without 5 μg/mL of polyinosine-polycytidylic acid (poly[I:C]) high molecular weight (HMW) (Invitrogen, USA) for 4 h. Then, the total RNA of the HD11 cells was extracted using TRIzol reagent (Thermo Fisher Scientific, USA) according to the manufacturer’s protocol. The cDNA was then synthesized using 2 μg of RNA with the RevertAid first-strand cDNA synthesis kit (Thermo Fisher Scientific, USA) according to the manufacturer’s protocol.

### Quantitative real-time polymerase chain reaction for target genes

Primers for quantitative real-time polymerase chain reaction (qRT-PCR) were designed using Primer-BLAST (https://www.ncbi.nlm.nih.gov/tools/primerblast/) and synthesized by Genotech (Daejeon, Korea) (Table 1). qRT-PCR was performed using Dyne qPCR 2X PreMIX (Dyne Bio, Seongnam, Korea) according to the manufacturer’s protocol with the CFX Connect Real-Time PCR Detection System (Bio-rad, Hercules, CA, USA). Chicken glyceraldehyde-3-phosphate dehydrogenase (*GAPDH*) was used for internal reference. The relative quantification of mRNA expression levels was calculated using the 2^–ΔΔCt^ method [26].

### Analysis of miRNA expression by qRT-PCR

To quantify miRNA expression, cDNA was synthesized using 2 μg of RNA with the Mir-X miRNA First-Strand Synthesis Kit (TAKARA Bio Inc., Otsu, Shiga, Japan) according to the manufacturer’s protocol. The forward primers of gga-miR-20a-5p, 5′-TAAAGTGCTTATAGTGCAGGTAG -3′, and small nuclear ribonucleoprotein polypeptide A (U1A), 5′-CTGCATAATTTGTGGTAGTGG -3′, were synthesized by Genotech (Korea). The miRNA expression level was evaluated using the Mir-X miRNA qRT-PCR TB Green Kit (TAKARA Bio Inc., Japan) with the CFX Connect Real-Time PCR Detection System (Bio-rad, USA). The miRNA expression level was calculated using the 2^–ΔΔCt^ method with U1A for internal reference.

### Luciferase reporter assay

For vector construction, the 3′-UTR of chicken interferon gamma receptor 2 (IFNGR2, NM_001008676), mitogen-activated protein kinase 1 (MAPK1, XM_015275131), mitogen-activated protein kinase kinase kinase 5 (MAP3K5, XM_015 284184), and mitogen-activated protein kinase kinase kinase 14 (MAP3K14, NM_001030927), including the binding site of miR-20a-5p, were amplified using wild type (WT) primers and mutant (Mut) primers (Table 1). The amplified PCR products of WT- and Mut-IFNGR2, MAPK1, MAP3K5, and MAP3K14 were cloned into a pMIR-REPORT luciferase vector (Thermo Fisher Scientific, USA). For the luciferase reporter assay, 1.2×10^6^ cells/well of the chicken fibroblast cell line DF-1 were seeded in a 6-well plate (SPL, Korea) containing 2.0 mL of culture medium and co-transfected with 40 pmol of miR-20-5p or miR-NC, 900 ng of WT or Mut plasmids, and 100 ng of the pMIR-REPORT β-gal control vector using the Lipofectamine 2000 Transfection Reagent (Thermo Fisher Scientific, USA) according to the manufacturer’s protocol. After 24 h of transfection, luciferase activity was detected using the Luciferase Assay System (Promega, Madison, WA, USA) and β-galactosidase activity was detected using the β-galactosidase enzyme assay system (Promega, USA) for normalization of transfection efficiency.

### Cell proliferation assay

HD11 cells (6.0×10^5^ cells/well) were plated in 24-well plates (SPL, Korea) containing 0.5 mL of exosome-depleted RPMI 1640 medium and transfected with exosomal gga-miR-20a-5p or miR-NC according to the method described above. After 44 h of transfection, cells were incubated with or without 5 μg/mL of poly(I:C) HMW (Invivogen, San Diego, CA, USA) for 4 h. Then, cell proliferation was measured using the Cell Counting Kit-8 (Enzo Life Science, Oyster Bay, NY, USA) according to the manufacturer’s protocol.

### Statistical analysis

Data are presented as the mean±standard error of the mean of three independent experiments. Statistical analyses were performed using the IBM SPSS software (SPSS 26.0; IBM, Chicago, IL, USA). Statistical comparison between two groups were evaluated using Student’s *t*-test. Statistical significance is defined when p-values are less than 0.05.

## RESULTS

### Characterization of isolated exosomes

**Figure 1:**
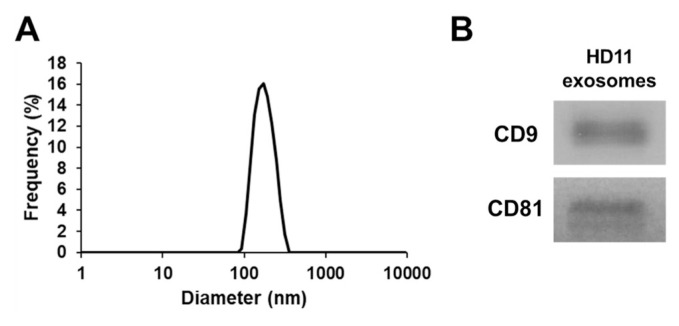
Characterization of exosomes. (A) Particle size distribution of exosomes in the chicken macrophage cell line HD11 measured using a nanoparticle analyzer. (B) Detection of exosomal markers, CD9 and CD81, by western blotting.

Exosomes were isolated from the cell culture supernatant of HD11 cells. The evaluated average size of the exosomes was 132.0 nm and the size range was from 82.33 to 279 nm (Figure 1A). The exosomal markers CD9 and CD81 were detected by western blotting (Figure 1B).

### Exosomal gga-miR-20a-5p directly targets the *IFNGR2*, *MAPK1*, *MAP3K5*, and *MAP3K14* genes

**Figure 2:**
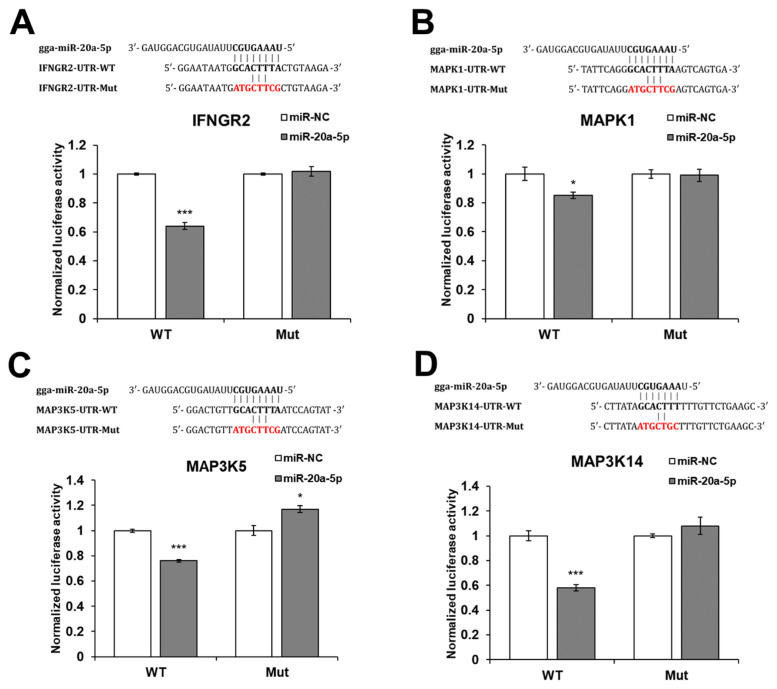
Luciferase reporter assay and potential binding site of gga-miR-20a-5p in target genes. For the luciferase reporter assay, the wild type (WT) or mutant (Mut) 3′-untranslated region (UTR) of (A) IFNGR2, (B) MAPK1, (C) MAP3K5, and (D) MAP3K14 were cloned into pMIR-REPORT luciferase vectors and co-transfected with mimic miR-20a-5p or negative control microRNA (miRNA) (miR-NC) in DF-1 cells by lipofectamine. IFNGR2, interferon gamma receptor 2; MAPK1, mitogen-activated protein kinase 1; MAP3K5, mitogen-activated protein kinase kinase kinase 5; MAP3K14, mitogen-activated protein kinase kinase kinase 14. * p<0.05 and *** p<0.001.

The gga-miR-20a-5p target genes were predicted using miRDB ( http://mirdb.org/) and TargetScan ( https://www.targetscan.org/vert_80/). Then, *IFNGR2*, *MAPK1*, *MAP3K5*, and *MAP3K14* genes were selected as candidate targets of gga-miR-20a-5p. To validate the target genes, a luciferase reporter assay was conducted by transfecting DF-1 cells with the WT or Mut 3′-UTR of IFNGR2, MAPK1, MAP3K5, or MAP3K14, along with mimic gga-miR-20a-5p or miR-NC. The luciferase activity of the cells transfected with WT IFNGR2, MAPK1, MAP3K5, and MAP3K14 was significantly reduced by miR-20a-5p compared with miR-NC, but the luciferase activity of the cells transfected with Mut IFNGR2, MAPK1, MAP3K5, and MAP3K14 was not reduced by miR-20a-5p (Figure 2).

**Figure 3:**
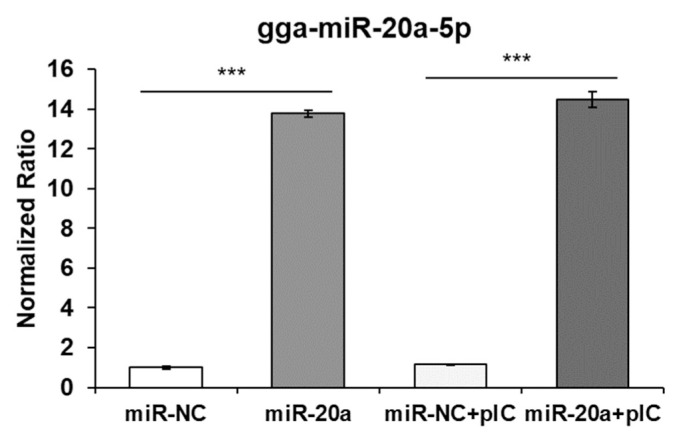
MicroRNA (miRNA) delivery efficiency using exosomes. HD11 cells were transfected with exosomes containing gga-miR-20a-5p or negative control miRNA (miR-NC). Then, miRNA delivery efficiency was evaluated by quantitative real-time polymerase chain reaction (qRT-PCR). Relative quantitation data are represented as mean±standard error of the mean (SEM) normalized to U1A using the 2^−ΔΔCt^ method. Data are expressed as mean±SEM of three independent experiments. *** p<0.001.

**Figure 4:**
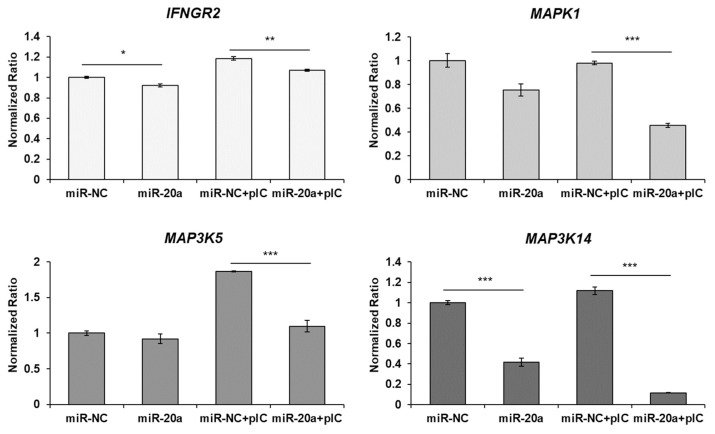
Exosome-mediated delivery of miR-20a-5p repressed target genes *in vitro*. HD11 cells were transfected with gga-miR-20a-5p or negative control microRNA (miRNA) (miR-NC) containing exosomes. After 44 h of transfection, cells were incubated with or without 5 μg/mL poly(I:C) for 4 h. Then, expression of the target genes, *IFNGR2*, *MAPK1*, *MAP3K5*, and *MAP3K14*, was evaluated by quantitative real-time polymerase chain reaction (qRT-PCR). Data are expressed as the mean±standard error of the mean (SEM) and are representative of three independent experiments. *IFNGR2*, interferon gamma receptor 2; *MAPK1*, mitogen-activated protein kinase 1; *MAP3K5*, mitogen-activated protein kinase kinase kinase 5; *MAP3K14*, mitogen-activated protein kinase kinase kinase 14. * p<0.05, ** p<0.01, *** p<0.001.

After transfection of the mimic gga-miR-20a-5p into the HD11 cells using exosomes, the transfection efficiency was evaluated by qRT-PCR (Figure 3). The expression of miR-20a-5p was significantly increased by transfection of miR-20a-5p using exosomes. Moreover, the expression of *IFNGR2*, *MAPK1*, *MAP3K5*, and *MAP3K14* was evaluated (Figure 4). The expressions of *IFNGR2* and *MAP3K14* were significantly downregulated in HD11 cells transfected by exosomal miR-20a-5p and poly(I:C)-stimulated exosomal miR-20a-5p-transfected HD11 cells. The expressions of *MAPK1* and *MAP3K5* were significantly downregulated in only poly(I:C)-stimulated HD11 cells transfected with exosomal miR-20a-5p.

### Exosomal miR-20a-5p represses the expression of interferon-stimulated genes and proinflammatory cytokines

**Figure 5:**
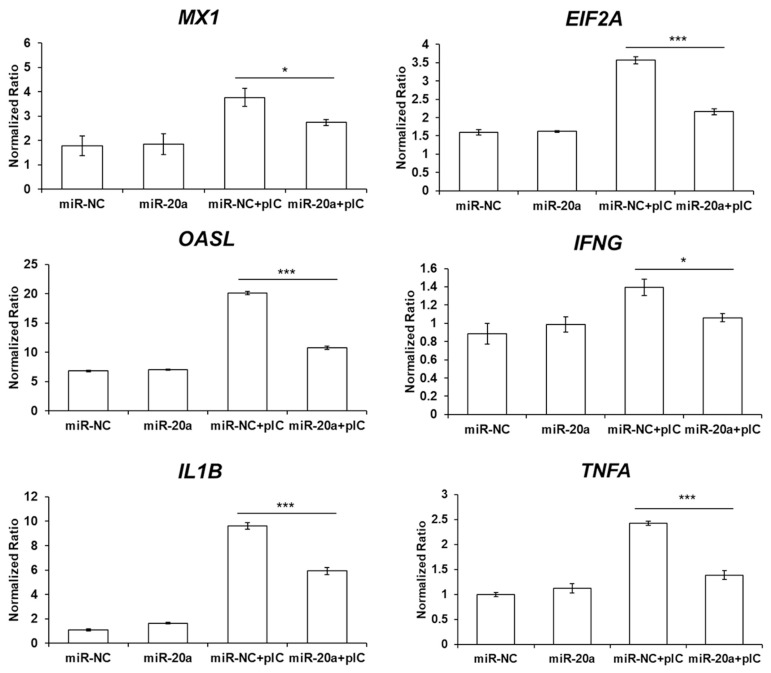
Exosome-mediated delivery of miR-20a-5p downregulated the expression of interferon-stimulated genes and proinflammatory cytokines. HD11 cells were transfected with gga-miR-20a-5p or negative control microRNA (miRNA) (miR-NC) containing exosomes. After 44 h of transfection, cells were incubated with or without 5 μg/mL of poly(I:C) for 4 h. Then, expressions of interferon stimulated genes (*MX1*, *EIF2A*, and *OASL*) and proinflammatory cytokines (IFN-γ, IL-1β, and TNF-α) were evaluated by quantitative real-time polymerase chain reaction (qRT-PCR). Data are expressed as the mean±standard error of the mean (SEM) and are representative of three independent experiments. *MX1*, MX dynamin like GTPase 1; *EIF2A*, eukaryotic translation initiation factor 2A; *OASL*, oligoadenylate synthase-like; IFN-γ, interferon gamma; IL-1β, interleukin-1beta; TNF-α, tumor necrosis factor alpha. * p<0.05 and *** p<0.001.

Because interferon (IFN)-γ induces interferon-stimulated gene (ISG) transcription and stimulates the expression of proinflammatory cytokines through the MAPK signaling pathway, the expression of ISGs and proinflammatory cytokines was evaluated by qRT-PCR (Figure 5). The expressions of ISGs, such as MX dynamin like GTPase 1 (*MX1*), eukaryotic translation initiation factor 2A (*EIF2A*), and oligoadenylate synthase-like (*OASL*) were downregulated in poly(I:C)-stimulated HD11 cells transfected by exosomal miR-20a-5p compared to cells transfected by exosomal miR-NC. Moreover, the expressions of proinflammatory cytokines, such as IFN-γ, IL-1β, and TNF-α, were downregulated in poly(I:C)-stimulated HD11 cells transfected by exosomal miR-20a-5p compared to those of the poly(I:C)-stimulated cells transfected by exosomal miR-NC.

### Exosomal miR-20a-5p increases cell proliferation

**Figure 6:**
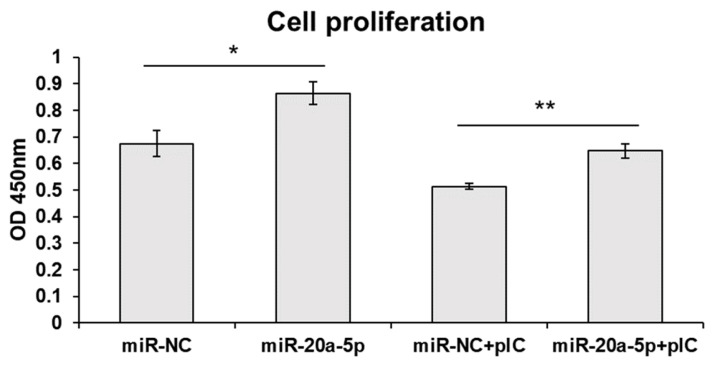
Exosome-mediated delivery of miR-20a-5p increased cell proliferation. HD11 cells were transfected with gga-miR-20a-5p or negative control microRNA (miRNA) (miR-NC) containing exosomes. After 44 h of transfection, cells were incubated with or without 5 μg/mL of poly(I:C) for 4 h. Then, cell proliferation was measured by the Cell Counting Kit-8 assay. Data are expressed as the mean±standard error of the mean and are representative of three independent experiments. * p<0.05 and ** p<0.01.

Since MAP3K5 and MAP3K14 are signaling molecules of apoptosis, we measured cell proliferation after exosome-mediated transfection of miR-20a-5p. Cell proliferation was increased by transfection with exosomal miR-20a-5p only and exosomal miR-20a-5p with poly(I:C) stimulation (Figure 6).

## DISCUSSION

In the present study, we delivered gga-miR-20a-5p via exosomes derived from a chicken macrophage cell line and identified the gene regulatory functions of gga-miR-20a-5p, which repressed the expression of *IFNGR2*, *MAPK1*, *MAP3K5*, and *MAP3K14*, in chicken macrophage cells.

MiR-20a-5p is a known regulator of immune responses. For example, miR-20a-5p was downregulated during liver fibrosis and transfection of miR-20a-5p reduced the production of the proinflammatory cytokines IL-6, TNF-α, IL-18, and IFN-γ in a murine hepatoma cell line by targeting transforming growth factor beta 2 (*TGFB2*) [27]. MiR-20a-5p is also upregulated in certain cancers, such as thyroid cancer [28], gastric cancer [29], breast cancer [30], and cervical cancer [31], by inducing cancer cell proliferation and inhibiting cell apoptosis. On the other hand, miR-20a-5p is normally downregulated in infectious and proinflammatory environments, such as tuberculosis [20], liver fibrosis [27], and rheumatoid arthritis [19], by stimulating proinflammatory cytokine production and apoptosis. In our previous study, the expression of exosomal miR-20a-5p was downregulated in H5N1-infected chickens [22]. Therefore, we predicted the target genes and validated the regulatory functions in chicken macrophages.

In this study, we stimulated chicken macrophages with poly(I:C) after transfection with exosomal miR-20a-5p. Macrophages as antigen presenting cells have crucial immune functions, such as phagocytosis of pathogens and production of cytokines and chemokines [32]. Moreover, poly(I:C) is a viral like double-stranded RNA (dsRNA) that binds to TLR3, RNA helicases, retinoic acid-inducible gene-I, and melanoma differentiation-associated protein 5. Upon recognition, the transcription of pro-inflammatory cytokines and interferons are activated by the transcription factors such as interferon regulatory factor-3 (IRF-3), active protein-1 (AP-1), and nuclear factor kappa-light-chain-enhancer of activated B cells (NF-κB) [33,34].

*IFNGR2*, *MAPK1*, *MAP3K5*, and *MAP3K14* were predicted as candidate target genes of gga-miR-20a-5p. In the canonical signaling pathway, after binding of IFN-γ and its receptors (*IFNGR1* and *IFNGR2*), phosphorylation of signal transducer and activator of transcription 1 (STAT1) by the Janus kinase 1 (JAK1) and Janus kinase 2 (JAK2) proteins induces STAT1 dimerization and translocation into the nucleus to activate ISG transcription [35]. In the non-canonical signaling pathway, IFN-γ activates various molecules, such as extracellular signal-regulated kinase (ERK), phosphoinositide 3-kinase (PI3K), proline-rich tyrosine kinase 2 (Pyk2), nuclear factor kappa-light-chain-enhancer of activated B cells (NF-κB), and ribonuclease A family 1 (Raf1). [36]. In this study, we identified that exosomal miR-20a-5p repressed the expression of *IFNGR2* and ISGs, such as *MX1*, *EIF2A*, and *OASL*, were downregulated in poly(I:C)-stimulated HD11 cells by exosomal miR-20a-5p. Type II IFNs, such as IFN-γ stimulate the phosphorylation of STAT1 by binding to IFNGR1 and IFNGR2. Then, gamma-activated sequences are activated by phosphorylated STAT1 and proinflammatory genes such as IL-1β and ISGs are induced [37]. Therefore, we suggest that the repressed expression of *IFNGR2* by exosomal miR-20a-5p decreased ISG transcription following the inhibited IFN-γ signal. The other candidate target genes, *MAPK1* (*ERK2*), *MAP3K5* (*ASK1*), and *MAP3K14* (*NIK*), are MAPK signaling pathway molecules (KEGG ID; gga04010). Moreover, *MAP3K5* and *MAP3K14* are also related to the apoptosis pathway (KEGG ID; gga04210). The TLR3 signaling pathway is known to activate the MAPK signaling pathway by inducing proinflammatory cytokine expression [38,39] and apoptosis [40]. Furthermore, the expression of TNF-α, a critical inflammatory cytokine responsible for necrosis or apoptosis, is also stimulated by the TLR3 signaling pathway [41,42]. In this study, we demonstrated that the expression of *MAPK1*, *MAP3K5*, and *MAP3K14* was downregulated by exosomal miR-20a-5p (Figures 2 and 4). In addition, the proliferation of chicken macrophage cells was increased by transfection of exosomal miR-20a-5p only and exosomal miR-20a-5p with poly(I:C) stimulation compared with transfection using exosomal miR-NC (Figure 6). Moreover, the expression of TNF-α was downregulated in poly(I:C)-stimulated macrophages by transfection of exosomal miR-20a-5p (Figure 5). Therefore, we suggest that the repressed expression of the MAPK signaling pathway molecules, *MAPK1*, *MAP3K5*, and *MAP3K14*, reduced TNF-α expression. Moreover, along with TNF-α reduction, the repressed expression of the apoptotic pathway molecules, *MAP3K5* and *MAP3K14*, increased the proliferation of chicken macrophages.

HPAIV H5N1 infection induces a massive production of proinflammatory cytokines, called a cytokine storm, which causes multiple organ dysfunction syndrome, acute respiratory distress syndrome, and sudden death [43–46]. Moreover, the MAPK signaling pathway is an important mediator of cytokine production against influenza A virus infection [47]. Therefore, we expected that the exosome-mediated delivery of gga-miR-20a-5p to H5N1-infected chicken cells could regulate proinflammatory cytokine expression by targeting the MAPK signaling pathway molecules, *MAPK1*, *MAP3K5*, and *MAP3K14*, and inhibit cell death by targeting the apoptosis signaling pathway molecules, *MAP3K5* and *MAP3K14*.

## CONCLUSION

In the present study, we evaluated gga-miR-20a-5p functions by delivery of exosomes into chicken macrophage cell lines. The expression of the candidate target genes, *IFNGR2*, *MAPK1*, *MAP3K5*, and *MAP3K14*, was repressed by exosomal miR-20a-5p. Moreover, the expression of ISGs and proinflammatory cytokines was downregulated by exosomal miR-20a-5p targeting IFNGR2 and MAPK signaling pathway molecules (MAPK1, MAP3K5, and MAP3K14). Cell proliferation was increased by the exosomal miR-20a-5p targeting the apoptosis pathway molecules (MAP3K5 and MAP3K14). However, although we identified repressed expression of miR-20a-5p target genes via a luciferase reporter assay and qRT-PCR, protein expression levels of the target genes were not measured because there are no commercially available antibodies against these chicken proteins. Moreover, additional studies such as evaluation of macrophage phenotypic changes and antiviral activity would be helpful in clarifying these results and conclusions, so continuous research on exosomal miR-20a-5p is needed. Taken together, the exosome-mediated delivery of gga-miR-20a-5p will help to maintain immune homeostasis against virus infections like HPAIV H5N1 by repressing the expression of proinflammatory cytokines and cell death.

## Figures and Tables

**Table 1:** Sequences of primers used for quantitative real-time polymerase chain reaction analysis

Gene	F/R	Primer sequences (5′–3′)	Accession No.
*GAPDH*	F	TGCTGCCCAGAACATCATCC	NM_204305
	R	ACGGCAGGTCAGGTCAACAA	
*IFNGR2*	F	TGAGTGCAGTTTCCTCAAGT	NM_001008676.3
	R	GGCCCTATGGTAGTGTTTCT	
*MAPK1*	F	ACTCAACACCTCAGCAACGA	XM_015275131.2
	R	GTTTGAGGTCGCGATGAAGC	
*MAP3K5*	F	CATTTGCACTGAACAGACGA	XM_040667792.1
	R	CTTGTAAATGCGACCAACCA	
*MAP3K14*	F	ATGGAGATGTGAAAGCGGAA	NM_001397364.1
	R	ATAGTTCCCTGTGACCAAGC	
*MX1*	F	AGCCATAGAACAAGCCAGAA	NM_204609.1
	R	GGTACTGGTAAGGAAGGTGG	
*EIF2AK2*	F	TCAGTCTGAGTCATGGGGTA	XM_015283611.3
	R	AGGTGCCAATACTCTTCTGG	
*OASL*	F	TCAAGACCGTCAAGGGCG	NM_001397447.1
	R	GGACTGGTGATGCTGACTCC	
*IFNG*	F	AACAACCTTCCTGATGGCGT	NM_205149.1
	R	TGAAGAGTTCATTCGCGGCT	
*IL1B*	F	TGCCTGCAGAAGAAGCCTCG	NM_204524.1
	R	CTCCGCAGCAGTTTGGTCAT	
*TNFA*	F	CGCTCAGAACGACGTCAA	XM_040694843.2
	R	TCGTCCCACACCAACGAG	
*IFNGR2-WT*	F	CGAGCTCGGAATAATGGCACTTTACTGTAAGA	NM_001008676
*IFNGR2-Mut*	F	CGAGCTCGGAATAATGATGCTTCGCTGTAAGA	
*IFNGR2-R*	R	CCAAGCTTACAATGAGAAGGATTAAGTGCCAT	
*MAPK1-WT*	F	CGAGCTCTATTCAGGGCACTTTAAGTCAGTGA	XM_015275131
*MAPK1-Mut*	F	CGAGCTCTATTCAGGATGCTTCGAGTCAGTGA	
*MAPK1-R*	R	CCAAGCTTGATGGGTTCCTATGTGGCTATTAC	
*MAP3K5-WT*	F	CGAGCTCGGACTGTTGCACTTTAATCCAGTAT	XM_015284184
*MAP3K5-Mut*	F	CGAGCTCGGACTGTTATGCTTCGATCCAGTAT	
*MAP3K5-R*	R	CCAAGCTTCCATGAAATGAGCATCTGGTTAC	
*MAP3K14-WT*	F	CGAGCTCCTTATAGCACTTTTTTGTTCTGAAGC	NM_001030927
*MAP3K14-Mut*	F	CGAGCTCCTTATAATGCTGCTTTGTTCTGAAGC	
*MAP3K14-R*	R	CCAAGCTTGCGTTAATGCTGTAGGTGAAGTGT	

*GAPDH*, glyceraldehyde-3-phosphate dehydrogenase; *IFNGR2*, interferon gamma receptor 2; *MAPK1*, mitogen-activated protein kinase 1; *MAP3K5*, mitogen-activated protein kinase kinase kinase 5; *MAP3K14*, mitogen-activated protein kinase kinase kinase 14; *MX1*, MX dynamin like GTPase 1; *EIF2AK2*, eukaryotic translation initiation factor 2 alpha kinase 2; *OASL*, oligoadenylate synthase-like; IFNG, interferon gamma; IL1B, interleukin-1beta; TNFA, tumor necrosis factor alpha.
